# Fatigue Behavior of an AM50 Die-Casting Alloy Anodized by Plasma Electrolytic Oxidation

**DOI:** 10.3390/ma14247795

**Published:** 2021-12-16

**Authors:** Kwangmin Choi, Seungwon Kang, Heon Kang

**Affiliations:** 1 Department of Materials Science and Engineering, Yonsei University, 50 Yonsei-ro, Seodaemun-gu, Seoul 03722, Korea; ckm88wkd@yonsei.ac.kr; 2 Titanium Department, Korea Institute of Materials Science, 797 Changwon-daero, Seongsan-gu, Changwon-si 51508, Korea; kenser@kims.re.kr; 3 School of Advanced Materials Engineering, Kookmin University, 77 Jeongneung-ro, Seongbuk-gu, Seoul 02707, Korea

**Keywords:** magnesium alloy, die-casting, fatigue properties, AM50, corrosion properties

## Abstract

While an anodizing process is essential for magnesium alloys to be used under corrosive environments, it sometimes stimulates a fatigue fracture that initiates at the interface between the coating layer and the substrate. In this study, a plasma electrolyte oxidation (PEO) technique was employed to provide excellent adhesion between the anodizing layer and the AM50 die-cast by applying an extremely high dielectric discharge in an alkaline phosphate electrolyte, and its effect on corrosion and fatigue behaviors was investigated. The stress intensity factor at the fatigue limit was estimated to be 0.28 MPam^0.5^. The specimen anodized using the PEO technique exhibits enhanced strength and corrosion resistance compared to the unanodized counterpart. Furthermore, it shows a relative fatigue life in spite of the thick anodizing layer because the crack initiates from the interface, not from the pore near the interface.

## 1. Introduction

Magnesium (Mg) and its alloys are used as engineering materials, as they have superior physical and mechanical properties, such as the lowest density (d = 1.7 g/cm^3^) in metals, a high specific strength to weight ratio, high dimensional stability and good machinability, which make them an excellent choice for applications in the automotive, aerospace and electronics industries [1,2,3]. The use of Mg in the aforementioned industries can significantly decrease the weight of components without sacrificing the mechanical requirements. Compared with gravity casting, in high-pressure die casting (HPDC), the metal is injected into the die at high velocity and solidifies under applied pressure. This process enables the production of intricate components at a relatively low cost and high production rates [4]. Then, the die-casting technique is often used in the manufacture of Mg components.

Although Mg has a lot of advantages, there has been a strong limitation arising from the weak corrosion protection of the passive films when Mg alloys were utilized in actual conditions, particularly corrosive environments [5,6]. The protection of Mg alloys from external corrosion is of great importance. The anodic process of magnesium and its alloys is a dissolution of magnesium, and the cathodic process evolves hydrogen. In practice, Mg corrodes with an apparent valence of less than 2.0 and the amount of evolved hydrogen in corrosion sites increases with an increasing anodic polarization. The reactions of the processes are defined as follows [7]:

Mg → Mg^2+^ + 2e^−^ (1)

2H_2_O + 2e^−^ → 2OH^−^ + H_2_ (2)

2Mg^+^ + 2H_2_O → Mg^2+^ + Mg(OH)_2_ + H_2_ (3)

Mg^+^ + 2H^+^ + 3e →MgH_2_↑ (4)

To this end, several traditional anodizing methods such as electrochemical plating and anodizing have been applied to form anodizing films in order to improve the corrosion resistance [8,9]. However, the anodizing layers formed by the traditional anodizing processes cannot protect the Mg substrate enough because the growth rate of the anodizing layer is irregular and the anodizing layer at the surface is heterogeneously formed. Plasma electrolytic oxidation (PEO) is a new electrochemical surface treatment method that generates a plasma state by applying an extremely high voltage in a suitable electrolyte, allowing magnesium alloys to form oxide layers with ease [10,11,12,13].

When considering magnesium alloys as structural materials, an improvement of their corrosion fatigue resistance is strongly requested. Therefore, fatigue tests are certainly carried out because fatigue loading is the main cause of failure of anodized materials, and the resistance of anodized materials to fatigue loading (i.e., failure) is evaluated either by stress to the number of cycles to failure (S–N) behavior [14,15,16] or fatigue crack propagation (FCP) behavior [16,17,18]. Unfortunately, fatigue strength has generally been reported to be degraded after anodizing and this degradation becomes more significant as the thickness of the anodizing layer increases [15,19].

In this study, we employed the PEO technique to produce an anodizing layer on the AM50 alloy and investigated its effect on the mechanical and corrosion behaviors of the AM50 alloy. In particular, we examined the role of the morphology of the interface between the anodized layer and the Mg substrate on the fracture morphology and failure mechanics.

## 2. Materials and Methods

A commercial AM50 alloy (Mg-5 wt% Al-0.64 wt% Mn) was cast using a high-pressure die casting method. The specimens (ASTM E139) were mechanically polished using 1000 grit emery paper and were subsequently rinsed with deionized water. Next, the plates were ultrasonically cleaned in ethanol and then dried. The direct current plasma electrolytic oxidation (PEO) process was adapted to coat the plates using a glass vessel container as an electrolyte cell under an applied current density of 50 mA/cm^2^. Stainless steel was selected as a counter electrode. A stirring and cold-water jacket cooling system was installed to maintain the electrolyte at a constant temperature. The chemical compositions of the electrolytes used in this study consisted of 0.089 M KOH + 0.052 KF + 0.009 M K4P2O7. The microstructure examination was carried out using field emission scanning electron microscopy (SEM, JEOL, JSM-7001, Tokyo, Japan), and corrosion potential and current density was measured using a typical electrochemical test (AMETEK, Versa STAT 3, Berwyn, PA, USA). The tests were performed with a conventional three-electrode cell consisting of a carbon plate as a counter electrode and a Calomel electrode (AMETEK, Berwyn, PA, USA) as a reference electrode at a temperature of 25 ± 1 ℃ in 0.1 M NaCl. Fatigue strength tests for the unanodized and PEO-anodized specimens were carried out at a stress ratio of 1 and a frequency of 5 Hz using a servo-hydraulic fatigue machine. Uniaxial tension tests were carried out on the unanodized and PEO-anodized alloys under a constant crosshead speed at an initial strain rate of 10^−4^ s^−1^ at an ambient temperature.

## 3. Results

**Figure 1:**
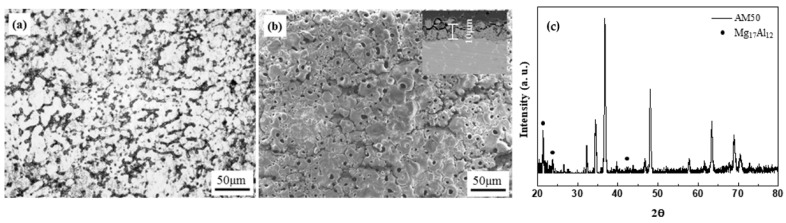
Microstructure of (**a**) the unanodized and (**b**) the anodized AM50 alloys. (**c**) XRD patterns of unanodized AM50 alloy.

The surface morphologies of the unanodized and the PEO-anodized specimens are shown in Figure 1. The existence of eutectic phases (Mg_17_Al_12_) in the unanodized specimen was determined with XRD analysis, as shown in Figure 1c. The eutectic phases are distributed at grain boundaries and the distance between the phases is about 15 μm. The PEO-anodized specimen shows a crater-like microstructure with some round shrinkage pores. The anodizing layer inserted in Figure 1b is about 10 μm in thickness.

**Figure 2:**
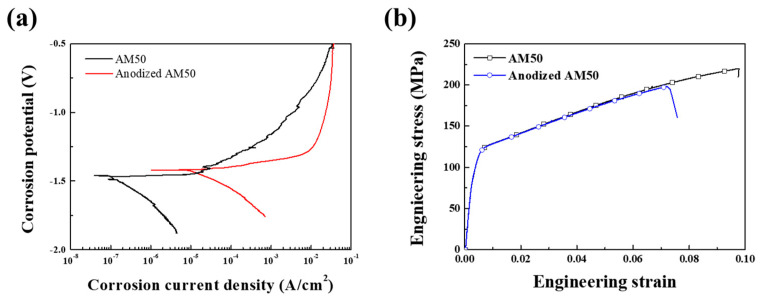
(**a**) Polarization curves, and (**b**) engineering stress-strain curves of the un-anodized and anodized AM50 alloys.

The corrosion properties of the unanodized and PEO-anodized AM50 alloys were also evaluated in 0.1 M NaCl solution, and the polarization curves are shown in Figure 2a. The related parameters fitted, such as the corrosion potential; the corrosion current density in the polarization curves of the unanodized and PEO-anodized AM50 alloys were obtained by using the cathode Tafel extrapolation, and the values of the parameters fitted are summarized in Table 1. The corrosion potential (*E_corr_*) of the PEO-anodized specimen is slightly increased since the anodizing layer is composed of magnesium oxide (*E_o_* = V) and the corrosion current density (*I_corr_*) is 100 times increased in comparison to the unanodized specimen. The corrosion rate as the penetration rate can be obtained using the corrosion current density. The conversion equations of the rate are as follows [20]:

(5) $$mpy\;:0.129\times (\left(EW\times I_{corr})/D\right)$$

(6) $$mm/yr\;:0.00327\times (\left(EW\times I_{corr})/D\right)$$

where *mpy*, *mm/yr*, *EW*, *I_corr_* and *D* are the mils penetration per year, the metric equivalent millimeter per year, equivalent weight, corrosion current density and density of the alloy, respectively. Since the *EW* of the AM50 alloy is 0.12, the mpy and *mm/yr* values of the die-casted AM50 alloy are converted to 0.066 and 0.0007 under 1M NaCl conditions. The values of the PEO-anodized alloy are also converted to 0.00169 and 0.000018 under the same conditions.

The mechanical properties of the die-casted specimens are shown at an ambient temperature in Figure 2b and summarized in Table 1. The values of the yield strength in the unanodized and the PEO-anodized specimens are 119 and 130 MPa, respectively. The yield strength of the PEO-anodized specimen is higher than that of the unanodized specimen because the elastic modulus of the anodizing layer is sufficiently higher than that of the substrate. However, due to the stress concentration at the interface between the anodizing layer and the substrate, the ultimate tensile strength (UTS) and the elongation to failure of the PEO-anodized specimen are lower than those of the unanodized specimen. The fracture toughness (*K*_1*c*_) was calculated using single edge notched tension tests. Specimens notched to form the surface and its length are defined as a. In the case of the *K*_1*c*_ single edge notched tension test, c is not only equal to 1/2a but also smaller than twice the thickness. When *c* is equal to a/2 and a/t is larger than 2, the *K*_1*c*_ is defined as follows [21]:

(7) $$K_{1c}=\left(P/B\sqrt{w}\right)f\left(a/w\right)$$

where *B* and *P* are the thickness of the specimen and the stress in the uniaxial direction, respectively *f(a/w)* is defined as follows:

(8) $$f\left(a/w\right)=\sqrt{2tan\left(\pi a/2w\right)}\times \frac{\left\{0.752+2.02\left(a/w\right)+0.37\left[1-sin{\left(\pi a/2w\right)}^{3}\right]\right\}}{cos\left(\pi a/2w\right)}\;$$

where *a* and *w* are the length of the notch and the width of the specimen, respectively. The calculated toughness value of the unanodized specimen is higher than that of the PEO-anodized specimen due to the reduced elongation to failure of the PEO-anodized specimen by the large elastic modulus difference between the anodizing layer and the substrate. However, the toughness of the PEO-anodized specimen is higher than that of pure Mg (10.2 MPam^0.5^) and the AM50 casting alloy (13.6 MPam^0.5^) [22].

**Figure 3:**
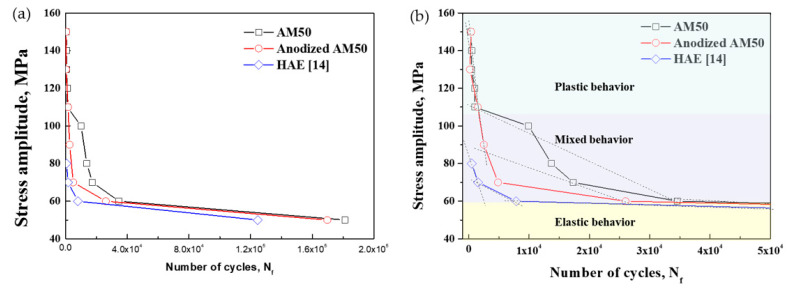
(**a**) S-N curves and (**b**) fatigue behavior for the unanodized and the anodized AM50 alloys.

In order to understand the fracture fatigue mechanism of the PEO-anodized AM50 die-casting alloy, the fatigue tests for the unanodized and the PEO-anodized specimens were carried out at an ambient temperature and the relationship between the stress amplitude and the number of cycles to failure (S-N curves) is shown in Figure 3a. Since the nucleation of a crack is sensitive to surface conditions, the thickness of the anodizing layer influences the fatigue nucleation life. Therefore, Figure 3a also shows the curve of the anodized specimen using the HAE process that was invented by Harry A. Evangelides [23], which generates the thin anodizing layer (about 1 μm in depth) [24]. The total fatigue life of the specimens is summarized in Table 2. As shown in Figure 3b, the number of cycles of all the specimens shows a tendency to decrease rapidly when the strength exceeds 60 MPa. When a strength of 60 MPa or less is applied, the cycles of all the specimens gradually increase. In the plastic deformation region (above 110 MPa), unanodized and PEO-anodized specimens show similar fatigue lifetimes. Since the slope in the range from 110 to 60 MPa is different from the slope in the plastic region, the range from 110 to 60 MPa shows mixed elasto-plastic behavior. At 60 MPa and below, the slope value decreases drastically because the region shows elastic behavior. The numbers of cycles at 60 MPa of the unanodized, the PEO-anodized and the HAE-anodized specimens are about 3.5 × 10^4^, 2.6 × 10^4^ and 0.7 × 10^4^ cycles.

The fatigue lifetime and the fatigue limit of the PEO-anodized (anodizing layer: 10 μm) and the HAE-anodized (anodizing layer: 1 μm) specimens are shorter and lower, respectively, compared to the unanodized specimen. Any voids, formed at the interface between the substrate and the coating layer during the anodizing process, may act as stress raisers and can stimulate fatigue crack propagation [25]. Although the effect of the thickness of the anodized layer at high-stress amplitudes is not clearly distinguished, the influence of the thickness has been proven at low-stress amplitudes. Moreover, the yield strength of the cyclic stress–strain curves of these specimens can be predicted to be the highest stress in the range of elastic behavior, and the strength of the monotonic stress–strain curve is also predicted to have the highest value in mixed behavior.

**Figure 4:**
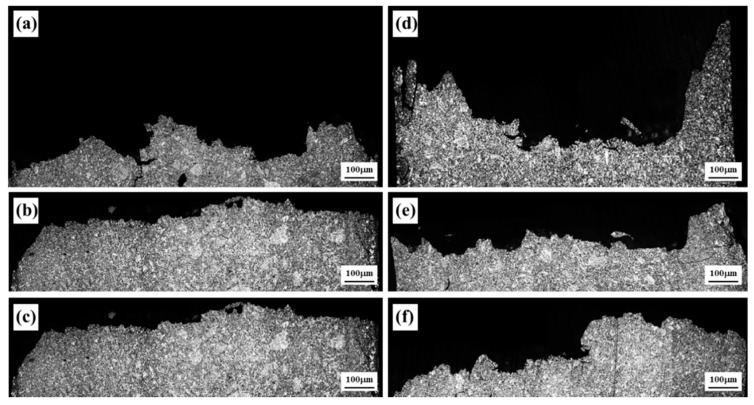
Microstructure of the cross-sectional observation of the fractured unanodized specimen at (**a**) 70, (**b**) 110 and (**c**) 150 MPa, and the fractured anodized specimens at (**d**) 70, (**e**) 110 and (**f**) 150 MPa.

The microstructure of the fractured specimens in a cross-section is observed and shown in Figure 4. The overall microstructures in a cross-section of the unanodized specimens at 70, 110 and 150 MPa are shown in Figure 4a–c respectively. The fracture images of the unanodized AM50 alloy reveal the typical fracture path that appears in the elastic region after the fatigue failure. During the fatigue testing of the alloy, the agglomerated internal coarse voids from microvoids initiate cracks when the stress concentration exceeds a critical value, while intermetallic compounds such as eutectic phases and inclusions also cause cracks. Therefore, the fracture path is affected by the size of the grains and the size and amount of intermetallics. The propagation of cracks is disturbed by grain boundaries and intermetallics. As revealed in Figure 4a, according to the above mechanism, the fracture path has meandered. As the applied load increases, the fracture paths of the unanodized specimens become flatter, as shown in Figure 4b,c. The microstructure of the PEO-anodized specimen at 70 MPa shows large meandering at the surface because the thick coating layer delays the crack propagation, as shown in Figure 4d. The fracture paths of the PEO-anodized specimens at 110 and 150 MPa also reveal the same phenomena as those of the unanodized specimens when the load is increased.

**Figure 5:**
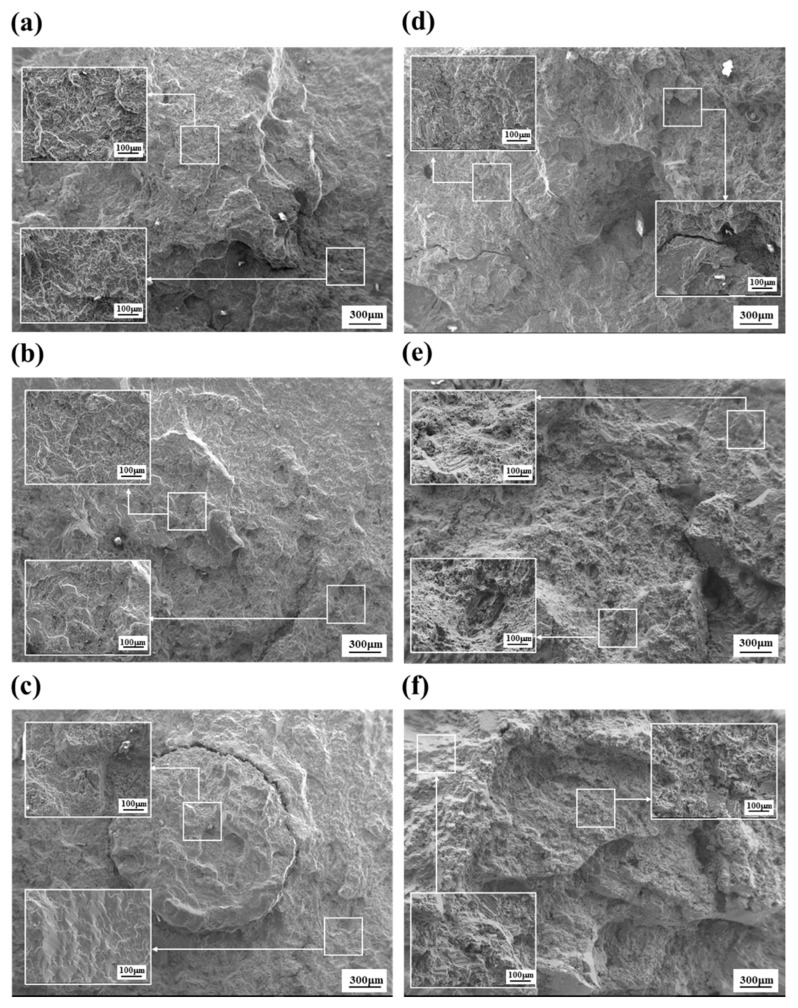
SEM images of the fractured unanodized specimen at (**a**) 70 (**b**) 110 and (**c**) 150 MPa, and the fractured anodized specimens at (**d**) 70 (**e**) 110 and (**f**) 150 MPa. Magnified images are in rectangular boxes in each image.

Figure 5a–c show the fractured surface images of the unanodized specimens at 70, 110 and 150 MPa, respectively. The images of the PEO-anodized specimens are also shown in Figure 5d–f. At 70 MPa, the cup-and-cone fracture surfaces caused by microvoids in both the unanodized and PEO-anodized alloys are revealed. However, the fracture surface of the unanodized specimens at 110 MPa shows the crack to have a circular shape (Figure 5b) and the shape is conspicuously revealed when the load is increased (Figure 5c). The crack shape is explained by the microstructure of the die-casting material, which has a large difference in microstructure between the surface and the center. The magnified images marked by rectangular boxes in the fracture surface of the unanodized specimens show that small cleavage facets are predominantly generated (Figure 5a). When the load is increased, as shown in Figure 5b,c, the fracture morphology changes to the large cleavage facets. However, when the load is increased, the size of the cleavage facet in the PEO-anodized specimens becomes slightly larger because the applied stress is dispersed by the relatively high strength thick anodizing layer, which reduces the fracture propagation rate.

**Figure 6:**
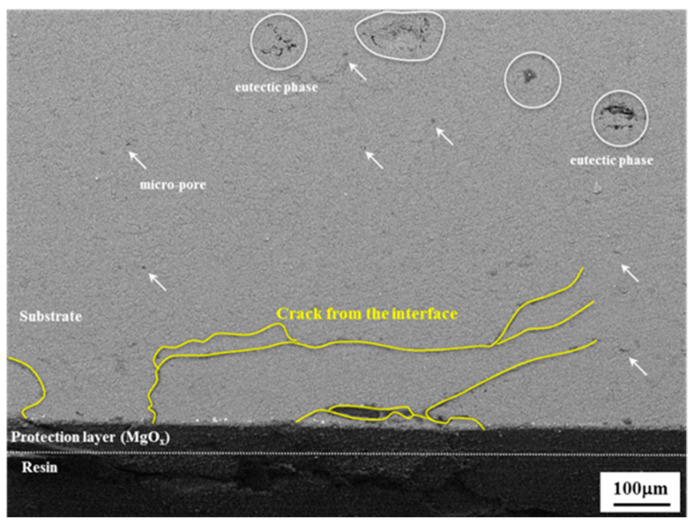
SEM images of the cross-sectional observation of the interrupted specimen at 110 MPa.

**Figure 7:**
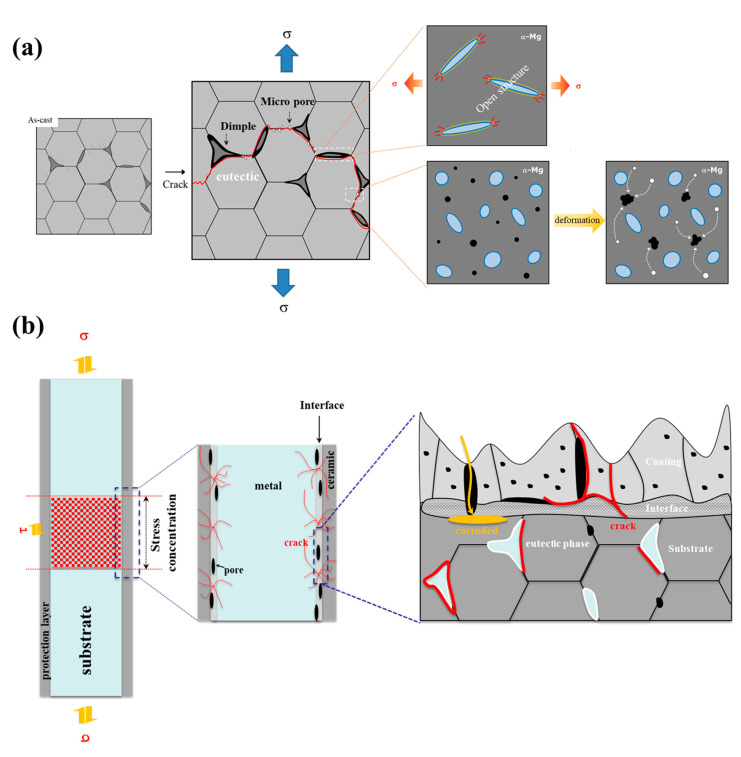
Fracture mechanism of the anodized alloy at (**a**) inner and (**b**) surface.

In Figure 6, the cross-sectional image of the interrupted specimen shows certain microcracks in the anodizing layer, while most of the microcracks remain to be small in size and some of them slightly increase up to failure. It is also obvious that the cracks initiate at the interface between the substrate and the anodizing layer and then grow to the substrate with a fatigue direction. After propagating a little through the anodizing layer, the cracks are arrested. Furthermore, meanwhile, the micro and macropores are generated at the eutectic phase due to the difference of elastic modulus between the matrix and the phase. Generally, fatigue life is essentially divided into the following two regions: crack nucleation and crack propagation. In the early stage (N_i_/N_f_ = 0.3), as illustrated in Figure 7a, the microcracks are nucleated [26]. However, in the same stage, the large cracks are dominantly observed in the material with the thin anodizing layer (less than 5 μm), while the microcracks are difficult to observe [27]. Furthermore, the initial crack is rarely formed at a large pore in the interface between the substrate and the anodizing layer when the thickness of the anodizing layer is over 5 μm. On the other hand, in the thin anodizing layer (less than 5 μm), fatigue cracks nucleate at the interface [28]. In this study, as illustrated in Figure 7b, since the anodizing layer of the PEO-anodized specimen is densely constructed with sufficient height, the fatigue cracks are generated from both the large pore and the interface. Since the self-passivation film of magnesium and its alloy is intrinsically very thin and unevenly formed, the formation rate of the anodizing layer is very irregular. Therefore, the fatigue life of most magnesium alloys with a thin anodizing layer through the HAE process is highly affected by cracks initiated in the pores of the anodizing layer. However, the fatigue life of the alloys with a thick anodizing layer, such as that generated by the PEO process, is greatly affected by cracks from the interface between the substrate and the anodizing layer. Although the processes that form a thick layer with a high rate of forming the anodizing layer generate many cracks in the layer, it is very difficult for the cracks to propagate through the ceramic layer, such as MgO or Mg(OH)_2._

To compare the fatigue properties in the specimens with anodizing layers of various thicknesses, the stress intensity factor of the PEO-anodized specimen was calculated. S. A. Khan and co-workers have suggested a model for an initial crack with the depth of the anodizing layer’s thickness [29]. With the crack size, the stress intensity factor range (∆*K*_1_) is calculated as follows [14]:

(9) $$\Delta K_{1}=H\Delta \sigma \sqrt{\pi b/E\left(k\right)F\left(b/t,b/a,a/W,\;\varphi \right)}$$

where *H* and ∆*σ* are a dimensionless parameter and stress range, respectively. *a* and *b* are the half diameter and thickness of an initial crack, respectively. *E(k)* is the Young’s modulus of the specimen according to the condition of the test; *t* and *W* are the thickness and the width of the specimen, respectively; *φ* is a geometric factor, which is a dimensionless function of the ratio of the crack’s length to width and the ratio of length to width.

**Figure 8:**
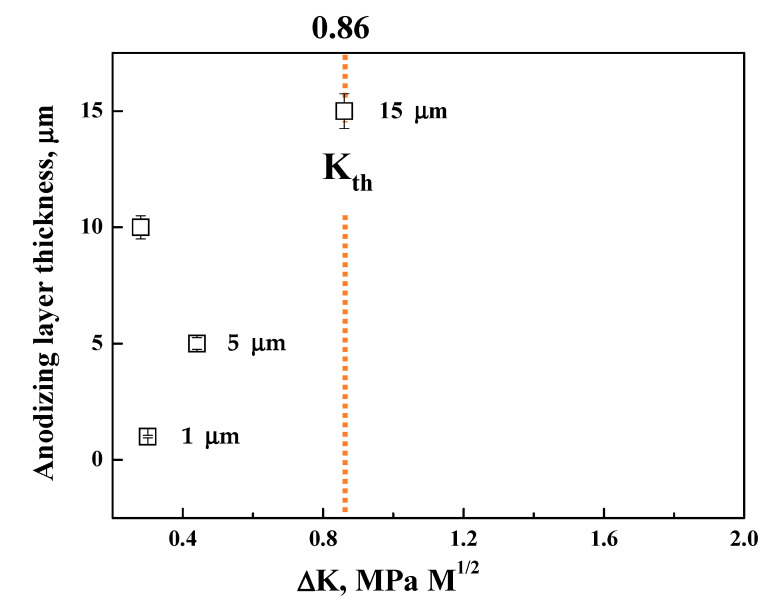
Relationship between the calculated stress intensity factor and the anodized layer thickness (1, 5, 15 μm: [33]).

The calculated results are shown in Figure 8. The ∆*K*_1_ value for the anodizing thickness of 15 μm is almost equal to the threshold stress intensity range, ∆*K_th_* (0.86 MPa√m) [30]. However, the ∆*K*_1_ value of the PEO-anodized specimen is close to the values of the specimens with thin anodizing layers. The small cracks are characterized by anomalous fatigue behavior and the small crack behavior is well expressed by the Kitagawa–Takahashi diagram indicating that the crack propagation threshold is a function of crack length [31]. The diagram is described by the following relationship:

(10) $$\Delta K_{th}=\Delta K_{tho}/\sqrt{1+l_{0}/l}$$

where ∆*K_tho_* is the crack size independent threshold stress intensity range for the long crack, and l and l0 are the crack length and the intrinsic crack length, respectively. Using Equation 10, the expression of the fatigue limit (Δσ_th_) can be obtained as follows:

(11) $$\Delta \sigma_{\mathrm{th}}=\Delta K_{tho}/k^{\prime }\sqrt{\pi \left(l+l_{0}\right)}$$

where *k*’ is the elastic concentration factor. The reduction rate of the fatigue limit with a change of the defect size is small when the defect size is smaller than the transition crack length [31]. Therefore, it is preferable for the thickness of the anodizing layer to be smaller than the thickness of the defect, which is smaller than the transition crack length. However, the Δσ_th_ value of the PEO-anodized specimen (Δσ_th_ = 74 MPa) is close to the value of the specimen with the thin anodizing layer (thickness of 1 μm, Δσ_th_ = 72 MPa [32]), although the long crack behavior was observed. The relatively long fatigue life of the PEO-anodized specimen is based on the fact that the crack initiates from the interface, not from the pore near the interface.

## 4. Conclusions

The fatigue tests and detailed observation of the commercial AM50 alloy anodized using the PEO method were carried out to investigate the effect of a thick anodized layer on the fatigue behavior of a die-casting alloy. In addition, the corrosion and mechanical properties of the unanodized and PEO-anodized alloys were also investigated. The main conclusions obtained are as follows:

(1) The die-casted AM50 alloy is anodized using the PEO method and a crater-like microstructure with some round shrinkage pores in the PEO-anodized alloy shown. The PEO-anodized AM50 Mg alloys with 10 μm in anodizing thickness exhibited ~110% enhanced strength and ~100 times enhanced corrosion current density. The corrosion potential of the PEO-anodized specimen is slightly increased, and the corrosion current density is 100 times increased compared to the unanodized specimen.
(2) The yield stress of the PEO-anodized specimens is 11 MPa higher than the unanodized alloy. The calculated toughness value of the unanodized alloy is higher than that of the PEO-anodized alloy at 13.6 MPam^0.5^ due to the decrease in the elongation to a failure caused by the thick anodized layer.
(3) The PEO-anodized AM 50 Mg alloys show good fatigue properties (i.e., Δ*K*_1_ value almost close to Δ*K_th_* (0.86 MPa√m) and Δσ_th_ of 74 MPa), the level of which corresponds to those of thin coating specimens.
(4) Since the anodizing layer of the PEO-anodized specimen is densely constructed, the 74 MPa of the Δσ_th_ value of the PEO-anodized alloy is close to the value of the thin layer anodized alloy. The cracks in the PEO-anodized alloy are predominantly generated at the interface between the substrate and the anodizing layer, resulting in a relatively long fatigue life.

## Figures and Tables

**Table 1:** Mechanical and Corrosion Properties of the un-Anodized and the Anodized AM50 Alloys.

	Mechanical Properties				Corrosion Properties	
	Yield Strength (MPa)	Ultimate Tensile Strength (MPa)	Elongation to Failure (%)	Fracture Toughness (MPam^1/2^)	Corrosion Potential (V)	Current Density (mA/mm^2^)
UnanodizedAM50	119	219	9.7	15.9	−1.41	7.9
Anodized AM50	130	197	7.2	14.4	−1.47	0.08

**Table 2:** Summary of Fatigue Life for the Alloys with Different Surface Treatments at 50 to 80 MPa.

		50 MPa	60 MPa	70 MPa	80 MPa
Total Fatigue life (cycle, N_f_)	Unanodized	181,211	34,584	17,318	13,711
	Anodized (depth: 10 mm)	169,758	25,993	7564	4868
	HAE (depth: 1 μm)	124,586	7846	1543	523

## Data Availability

Data available in a publicly accessible repository.
